# Loss of β-Ketoacyl Acyl Carrier Protein Synthase III Activity Restores Multidrug-Resistant Escherichia coli Sensitivity to Previously Ineffective Antibiotics

**DOI:** 10.1128/msphere.00117-22

**Published:** 2022-05-16

**Authors:** Yaoqin Hong, Jilong Qin, Anthony D. Verderosa, Sophia Hawas, Bing Zhang, Mark A. T. Blaskovich, John E. Cronan, Makrina Totsika

**Affiliations:** a Centre of Immunology and Infection Control, School of Biomedical Sciences, Faculty of Health, Queensland University of Technology, Brisbane, Queensland, Australia; b Centre for Superbug Solutions, Institute for Molecular Bioscience, University of Queensland, Brisbane, Queensland, Australia; c Department of Microbiology, School of Molecular and Cellular Biology, University of Illinois at Urbana-Champaign, Urbana, Illinois, USA; d Department of Biochemistry, School of Molecular and Cellular Biology, University of Illinois at Urbana-Champaign, Urbana, Illinois, USA; University of Rochester

**Keywords:** fatty acid biosynthesis, outer membrane permeability, antibiotic potentiation, multidrug resistance

## Abstract

Antibiotic resistance is one of the most prominent threats to modern medicine. In the latest World Health Organization list of bacterial pathogens that urgently require new antibiotics, 9 out of 12 are Gram-negative, with four being of “critical priority.” One crucial barrier restricting antibiotic efficacy against Gram-negative bacteria is their unique cell envelope. While fatty acids are a shared constituent of all structural membrane lipids, their biosynthesis pathway in bacteria is distinct from eukaryotes, making it an attractive target for new antibiotic development that remains less explored. Here, we interrogated the redundant components of the bacterial type II fatty acid synthesis (FAS II) pathway, showing that disrupting FAS II homeostasis in Escherichia coli through deletion of the *fabH* gene damages the cell envelope of antibiotic-susceptible and antibiotic-resistant clinical isolates. The *fabH* gene encodes the β-ketoacyl acyl carrier protein synthase III (KAS III), which catalyzes the initial condensation reactions during fatty acid biosynthesis. We show that *fabH* null mutation potentiated the killing of multidrug-resistant E. coli by a broad panel of previously ineffective antibiotics, despite the presence of relevant antibiotic resistance determinants, for example, carbapenemase *kpc2*. Enhanced antibiotic sensitivity was additionally demonstrated in the context of eradicating established biofilms and treating established human cell infection *in vitro*. Our findings showcase the potential of FabH as a promising target that could be further explored in the development of therapies that may repurpose currently ineffective antibiotics or rescue failing last-resort antibiotics against Gram-negative pathogens.

**IMPORTANCE** Gram-negative pathogens are a major concern for global public health due to increasing rates of antibiotic resistance and the lack of new drugs. A major contributing factor toward antibiotic resistance in Gram-negative bacteria is their formidable outer membrane, which acts as a permeability barrier preventing many biologically active antimicrobials from reaching the intracellular targets and thus limiting their efficacy. Fatty acids are the fundamental building blocks of structural membrane lipids, and their synthesis constitutes an attractive antimicrobial target, as it follows distinct pathways in prokaryotes and eukaryotes. Here, we identified a component of fatty acid synthesis, FabH, as a gate-keeper of outer membrane barrier function. Without FabH, Gram-negative bacteria become susceptible to otherwise impermeable antibiotics and are resensitized to killing by last-resort antibiotics. This study supports FabH as a promising target for inhibition in future antimicrobial therapies.

## INTRODUCTION

The outer membrane (OM) of Gram-negative bacteria is critical to their survival within harsh yet fluctuating environments (1). Unlike the canonical cytoplasmic membrane, the OM has an asymmetrical design, in which the inner leaflet is almost exclusively composed of phospholipids (PLs) while the outer leaflet is filled with the lipid A components of lipopolysaccharide (LPS) (2). Each LPS molecule carries anionic charges due to phosphate and carboxylate groups, which provides much of the OM rigidity through intermolecular interactions. The uniformly distributed LPS molecules on the outer leaflet of OM make it a potent barrier and guard the bacterium from harmful compounds (3). Moreover, each LPS molecule is capped by a hydrophilic core oligosaccharide, and in most cases, a terminal long-chain polysaccharide is also attached to the core oligosaccharide. In effect, this creates an additional stabilizing and protecting water-rich layer extending from the cell surface (4). For nutrient uptake, the otherwise impermeable OM houses a set of specialized porin proteins that allows solute exchange (3).

Over the past decades, the emergence of resistance to currently available antibiotics among many clinically important bacterial pathogens has been recognized as a major threat to global public health. In response to this challenge, the World Health Organization released a list of high-priority pathogens urgently requiring new antimicrobials (5, 6). Within the critical priority category of this list is the third-generation cephalosporin and/or carbapenem-resistant *Enterobacteriaceae* (including Escherichia coli, Klebsiella, *Serratia*, and Proteus). Many circulating pathogenic E. coli lineages are multidrug-resistant (MDR) and therefore remain susceptible to only a few available treatment options, which are fast diminishing (7, 8). One key contributor to antimicrobial resistance (AMR) in Gram-negative bacteria is their OM. Most effective antibiotics access their intracellular targets using OM-spanning hydrophilic porin channels, but this transport is restricted by both the chemical properties and the size of the permeant antibiotics. In the *Enterobacteriaceae* family, only small hydrophilic antibiotics (<600 kDa) can permeate the generalized porins (9, 10).

In bacteria, the essential acyl chains of PL and LPS are *de novo* synthesized by the highly conserved type II fatty acid synthesis pathway (FAS II) (11, 12). The structural heterogeneity of acyl chains present in membrane lipids can determine how bacteria respond to challenging and fluctuating environments (13–19). As seen in other essential pathways, FAS II has evolved specialized components, which possess overlapping biochemical activities and complex redundancies. Thus, several enzymes are redundant despite the essentiality of their biochemical activities. For example, in E. coli, three β-ketoacyl acyl carrier protein (ACP) synthases (KAS proteins)—FabB for KAS I activity (encoded by *fabB*), FabF for KAS II (encoded by *fabF*), and FabH for KAS III (encoded by *fabH*)—collectively take part in the Claisen condensation reactions to drive fatty acid (FA) biosynthesis (11). FabH initiates FA biosynthesis through condensing acetyl coenzyme A (CoA) with malonyl ACP (20), while FabB and FabF take overlapping roles in the subsequent polymerization steps (11). The associated substrate specificity has been reported in great detail by J. L. Garwin et al. (21).

The PL-LPS ratio, maintenance of membrane asymmetry, surface charge profiles of the surface exposed LPS, and chemical differences within both the PLs and LPS components collectively define the overall physicochemical and biological properties of the OM (22). As such, progress in understanding the OM may lead to valuable tactics to circumvent this formidable protective barrier and improve antibiotic cell entry in Gram-negative bacteria. In this work, we examined the role of redundant FAS II genes in maintaining the antimicrobial exclusion properties of the membrane barrier in clinically relevant uropathogenic E. coli (UPEC) strains, including a reference MDR isolate from the globally disseminated sequence type 131 (ST131) lineage (23, 24). We report that UPEC Δ*fabH* strains lacking KAS III activity display a severely defective membrane envelope and therefore are highly sensitive to antibiotic killing (up to 41-fold reduced MIC), even by otherwise ineffective drugs and while still harboring relevant AMR determinants. Moreover, this increased sensitivity held true in established biofilm eradication and the treatment of infected human bladder cell monolayers. Together, this work showcases FabH as a promising FAS II component that could be therapeutically targeted to rescue failing last-resort antibiotics and expand the range of currently available antibiotic treatments for Gram-negative infections.

## RESULTS

### *FabH* contributes to outer membrane barrier function in UPEC.

**FIG 1:**
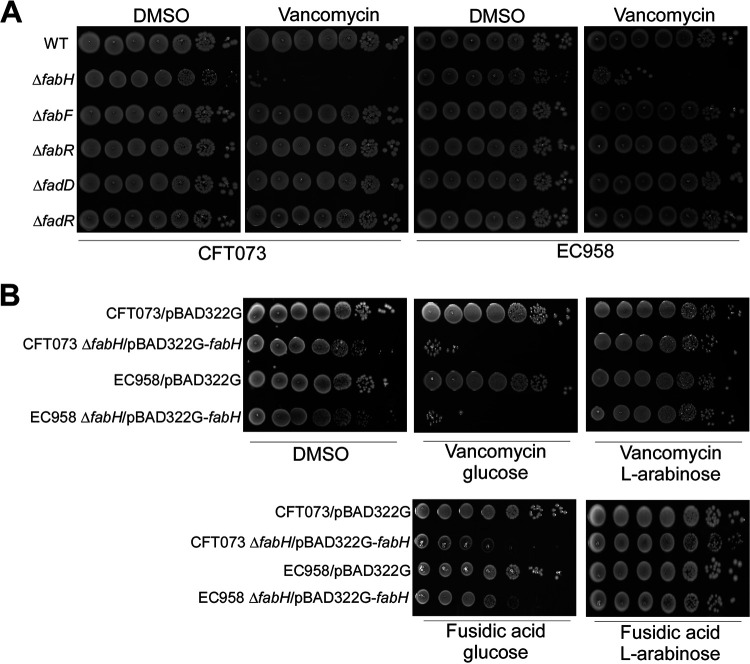
Screening UPEC FAS II mutants for membrane barrier defects using vancomycin. (A) CFT073 and EC958 WT and FAS II mutants were cultured on LB-Lennox agar containing 50 μg/mL vancomycin or DMSO carrier control. (B) Complementation of defective membrane barrier in CFT073 and EC958 Δ*fabH* using plasmid-borne *fabH* expressed under arabinose control. Overnight cultures normalized to an OD_600_ of 1.0 were serially diluted to 1E-6, and 5 μL of each dilution was spotted platted onto plates containing DMSO, 50 μg/mL vancomycin, or 100 μg/mL fusidic acid. Where appropriate, 1% d-glucose or 50 mM l-arabinose was supplemented to suppress or induce the expression of plasmid-encoded *fabH*, respectively. The plate images shown are representative of at least three independent experiments.

To study the involvement of the redundant genes in the FAS II pathway in the maintenance of the OM barrier, we used two model UPEC strains, CFT073 (a drug-sensitive reference pyelonephritis isolate) and EC958 (a reference MDR ST131 cystitis isolate), and constructed null mutants of the *fabH*, *fabF*, *fabR*, *fadR*, and *fadD* genes. Mutants were evaluated for OM defects by measuring their susceptibility to subinhibitory concentrations of vancomycin (25). UPEC Δ*fabF*, Δ*fabR*, Δ*fadR*, and Δ*fadD* mutants had growth similar to that of the wild type (WT) on LB-Lennox containing 50 μg/mL vancomycin, in both strains (Fig. 1). Like previous E. coli K-12 Δ*fabH* studies (26, 27), UPEC Δ*fabH* strains grew remarkably slower than the WT strains (Fig. S1), and this is reflected in the reduced colony size illustrated in Fig. 1A. Notably, despite the growth defect, the Δ*fabH* sample plated onto dimethyl sulfoxide (DMSO) carrier control plates contained a similar number of viable CFU as the optical density at 600 nm (OD_600_)-matched WT inoculum (Fig. 1A).

10.1128/msphere.00117-22.4 FIG S1 Growth of E. coli UPEC wild-type and Δ*fabH* strains in LB. Overnight cultures were diluted to an OD_600_ of 0.1 in LB-Lennox. Growth rates were tracked by changes in OD_600_ using CLARIOStar (BMG, Australia). The experiments were performed in triplicates, with the error bar indicative of SD values. Download FIG S1, TIF file, 0.6 MB. Copyright © 2022 Hong et al. 2022 Hong et al. https://creativecommons.org/licenses/by/4.0/ This content is distributed under the terms of the Creative Commons Attribution 4.0 International license.

**FIG 2:**
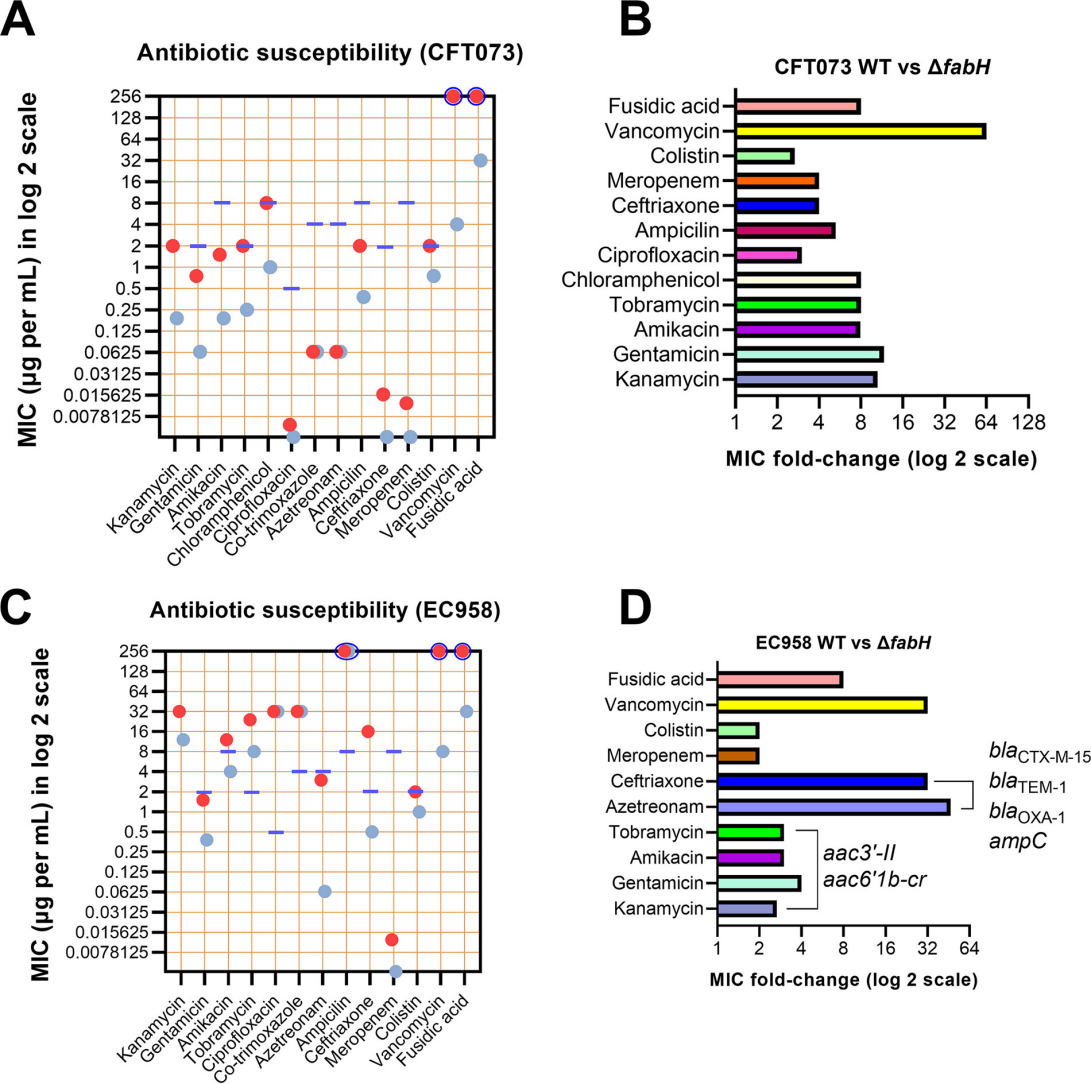
Loss of KAS III activity potentiates UPEC killing by a wide array of antibiotics. (A) Individual antibiotic MICs for CFT073 WT (red dots) and Δ*fabH* strains (blue dots). (B) Antibiotic MIC fold changes (improvement in potency) for the CFT073 Δ*fabH* mutant relative to the WT. (C) Individual antibiotic MICs for EC958 WT (red dots) and Δ*fabH* (blue dots). (D) Antibiotic MIC fold changes (improvement in potency) for the EC958 Δ*fabH* mutant relative to the WT. The purple lines in panels A and C mark current clinical resistance MIC cutoffs for each antibiotic as listed in the European Committee on Antimicrobial Susceptibility Testing tables (31). MIC values that exceeded the testing range of the Liofilchem MIC strips are indicated by blue circles that enclose the red/blue dot(s).

As expected, WT strains of EC958 and CFT073 survived low-dose vancomycin, a Gram-positive antibiotic that is normally ineffective against Gram-negative bacteria, as the OM prevents penetration to reach its peptidoglycan target. In contrast, CFU of the Δ*fabH* mutants were diminished by 5-logs when exposed to 50 μg/mL vancomycin (Fig. 1A). Likewise, we also observed similar increased sensitivity to fusidic acid, a hydrophobic antibiotic, which like vancomycin, is normally only effective against Gram-positive bacteria (Fig. 2B). We then introduced the *fabH* gene into the Δ*fabH* mutants on the low-copy-number and tightly controllable pBAD322G vector under P*ara* control (28). Intrinsic resistance to both vancomycin and fusidic acid was fully restored to WT levels upon induction of FabH expression, but not when transcription was catabolically repressed (Fig. 1B). We also noticed a similar susceptibility pattern in laboratory K-12 strains; thus, the cryptic OM of the Δ*fabH* strain is not restrictive to UPEC but generally applies to most, if not all, E. coli strains (data not shown).

### Loss of *FabH* promotes UPEC killing by several antibiotics.

We hypothesized that the defective OM allows the efficient penetration of antibiotics and potentiates their inhibitory effect. To assess this, we determined the MIC of Δ*fabH* strains to a wide range of antibiotics (Fig. 2 and Table S2). As expected, fusidic acid and vancomycin that cannot penetrate the Gram-negative cell envelope displayed an MIC of >256 μg mL^−1^ against CFT073 WT (Fig. 2A). In marked contrast, for CFT073 Δ*fabH*, the vancomycin MIC was reduced by >64-fold, whereas for fusidic acid, a >8-fold MIC reduction was observed (Fig. 2A and B). Antibiotics representing the β-lactam, aminoglycoside, phenicol, polymyxin, and quinolone/fluoroquinolone classes were also tested. A >8-fold increase in susceptibility to both aminoglycosides (kanamycin, gentamicin, amikacin, and tobramycin) and phenicol (chloramphenicol) was observed. Moreover, the MIC values of ceftriaxone, meropenem, colistin, and ciprofloxacin against the isogenic CFT073 Δ*fabH* mutant were further decreased by 2- to 4-fold (Fig. 2A and B), despite these antibiotics already being active at sub-μg per mL concentrations against the susceptible CFT073 strain. Interestingly, we found CFT073 to be phenotypically resistant to tobramycin, chloramphenicol, and colistin, although it lacks recognizable antibiotic-resistant determinants (29).

10.1128/msphere.00117-22.2 TABLE S2 Antibiotic MIC values against UPEC wild-type and Δ*fabH* strains (cells showing significant fold changes [>20-fold] in MIC are highlighted in gray). Download Table S2, DOCX file, 0.01 MB. Copyright © 2022 Hong et al. 2022 Hong et al. https://creativecommons.org/licenses/by/4.0/ This content is distributed under the terms of the Creative Commons Attribution 4.0 International license.

The restored susceptibility to three antibiotics (tobramycin, chloramphenicol, and colistin) in CFT073 Δ*fabH* prompted us to test if antibiotic efficacy could be restored against resistant strains. EC958 is an MDR clinical isolate of the globally disseminated ST131 lineage, carrying resistance genes to several antibiotic classes, including β-lactams (*bla*_CTX-M-15_, *bla*_TEM-1_, *bla*_OXA-1_, *bla*_CMY23_, and *ampC*), aminoglycosides (*aac3′-II* and *aac6'1b-cr*), and sulfonamides (*dhfrVII*, *aadA5*, and *sul1*), in addition to two chromosomal mutations (S83L and D87N mutations in the *gyrA* gene) that confer fluoroquinolone resistance (23, 24, 30).

We attributed the very high co-trimoxazole and ciprofloxacin MIC values (>32 μg/mL) of EC958 WT and Δ*fabH* to the sulfonamide and quinolone resistance determinants present in the strain (Fig. 2C). Like in CFT073, loss of *fabH* rendered EC958 susceptible to vancomycin (>32-fold change in MIC), fusidic acid (>8-fold), and colistin (2-fold) (Fig. 2D). Moreover, relative to the WT, EC958 Δ*fabH* was >2- to 4-fold more susceptible to all four tested aminoglycosides (note that for EC958 WT, the kanamycin MIC exceeded the test range of the MIC strip, so the reported fold change is likely an underestimate; Fig. 2C and D). Importantly, amikacin susceptibility was restored in the EC958 Δ*fabH* mutant (Fig. 2C and D), despite the presence of the aminoglycoside resistance genes (23, 30). We next compared the susceptibility of EC958 WT and Δ*fabH* strains to ampicillin, ceftriaxone, and aztreonam. As an extended-spectrum β-lactamase-positive (ESBL-positive) isolate, EC958 was found to be resistant to ceftriaxone (16 μg/mL) and nearly intermediate-resistant to aztreonam (3 μg/mL) (Fig. 2C). For EC958 Δ*fabH*, we observed enhanced susceptibility relative to the WT. The MIC values for ceftriaxone and aztreonam were reduced by 32-fold and ~47-fold, respectively (Fig. 2D). The extent of this MIC reduction rendered EC958 Δ*fabH* clinically susceptible to these otherwise ineffective antibiotics (with MICs well below the sensitivity values reported for *Enterobacterales* [31]).

**FIG 3:**
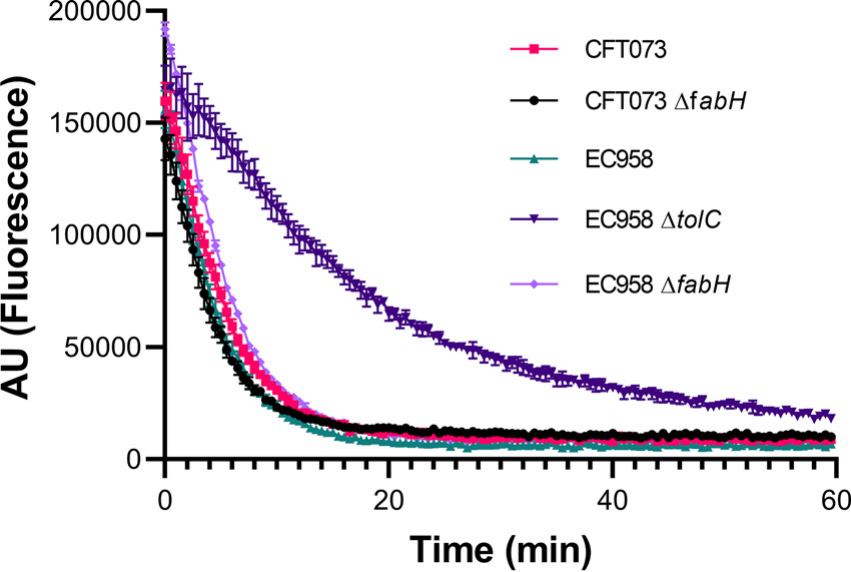
Efflux activity is unaffected in UPEC Δ*fabH* strains. Efflux kinetics of accumulated ethidium bromide were tracked in late-phase bacterial cultures by excitation at 525 nm and emission at 615 nm. Experiments were performed in biological triplicates, and average fluorescence readings (arbitrary units) taken every 30 s are plotted with error bars showing standard deviation (SD) values.

The localization and folding of outer membrane proteins can be impacted by compositional changes in the bacterial membrane envelope (see recent review by J. E. Horne et al. [32] for detailed information). Therefore, in addition to the severed permeability barrier, efflux pumps may be impacted in Δ*fabH* and, as such, partially contribute to the observed antibiotic hypersensitivity. We probed the efflux activity of WT and Δ*fabH* strains of both CFT073 and EC958, using intracellularly accumulated ethidium bromide (Fig. 3). A Δ*tolC* mutant was constructed in EC958 as an efflux-defective control. As expected, ethidium bromide efflux was significantly delayed in this mutant (Fig. 3). In contrast, ethidium bromide was rapidly expelled from both the WT control and the Δ*fabH* mutant (Fig. 3). We conclude that efflux activity is unaffected in UPEC Δ*fabH* strains.

### Loss of KAS III activity restores UPEC sensitivity to last-line carbapenems.

Intriguingly, unlike other antibiotics of the β-lactam class, the Δ*fabH* strains were only twice more susceptible to meropenem than the WT strains (Fig. 2D). Both UPEC strains lack genes for carbapenem resistance, so we introduced a *kpc2*-containing plasmid (medium copy pSU2718 vector [33]) into the WT and Δ*fabH* strains to directly determine the impact of losing KAS III activity on carbapenem resistance.

**FIG 4:**
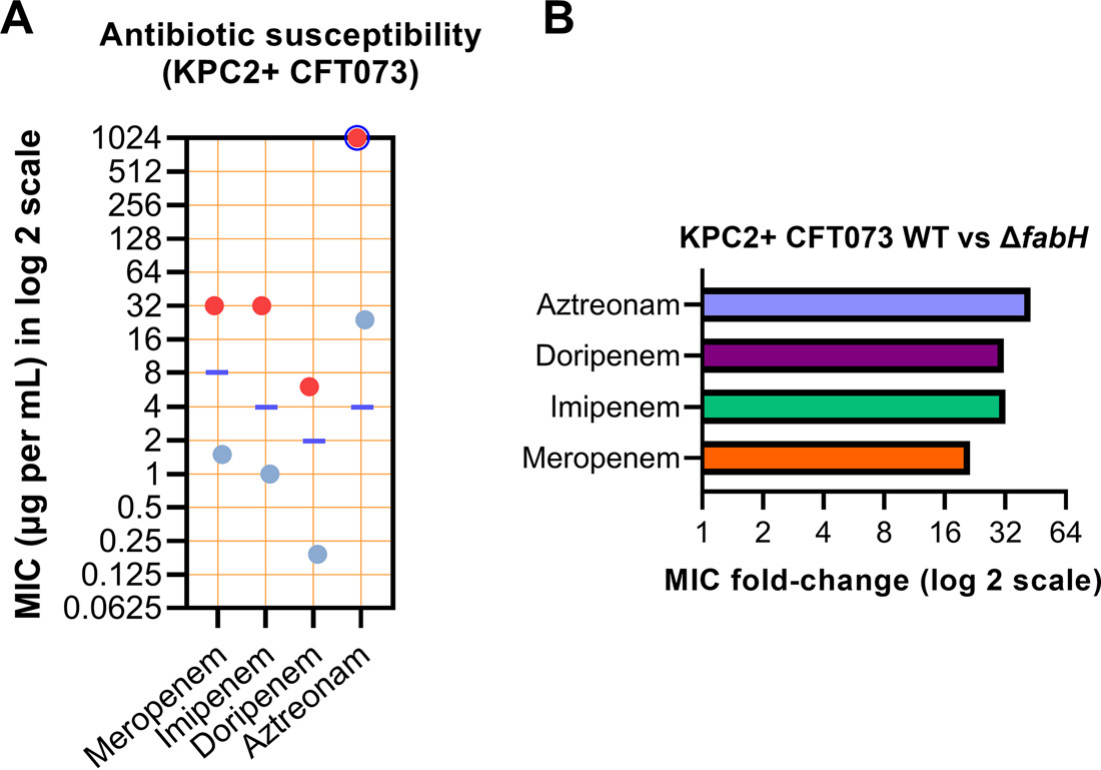
Carbapenemase-producing UPEC Δ*fabH* show restored susceptibility to carbapenem antibiotics. (A) MIC values for four carbapenem antibiotics tested against KPC-producing CFT073 WT (red dots) and isogenic Δ*fabH* (blue dots). The purple line indicates the latest current resistant MIC cutoff value for each antibiotic listed in the European Committee on Antimicrobial Susceptibility Testing tables (29). For MIC values that exceeded the testing range of the Liofilchem MIC strips, a blue circle is used to enclose the red/blue dot(s). (B) Carbapenem susceptibility MIC fold change (improvement in potency) for the KPC2+ CFT073 Δ*fabH* mutant relative to WT.

Remarkably, the KPC-producing CFT073 Δ*fabH* strain displayed a reduced meropenem MIC by more than 21-fold relative to the WT strain (Fig. 4 and Table S3). Similarly pronounced results were observed for three other carbapenem antibiotics, with the MIC of imipenem, doripenem, and aztreonam reduced by 32-fold, >31-fold, and >42-fold, respectively (Fig. 4B and Table S3). All tested carbapenems (other than aztreonam) regained clinical potency against the KPC-producing UPEC Δ*fabH* strain. Overall, the loss of KAS III activity rendered the resistant parent strain remarkably susceptible to last-line carbapenems, with MIC values 4- to 10.5-fold lower than the current European Committee on Antimicrobial Susceptibility Testing resistance cutoff values for *Enterobacterales* (31).

10.1128/msphere.00117-22.3 TABLE S3 Antibiotic MIC values against KPC2+ CFT073 wild-type and Δ*fabH* strains (cells showing significant fold changes [>20-fold] in MIC are highlighted in gray). Download Table S3, DOCX file, 0.01 MB. Copyright © 2022 Hong et al. 2022 Hong et al. https://creativecommons.org/licenses/by/4.0/ This content is distributed under the terms of the Creative Commons Attribution 4.0 International license.

**FIG 5:**
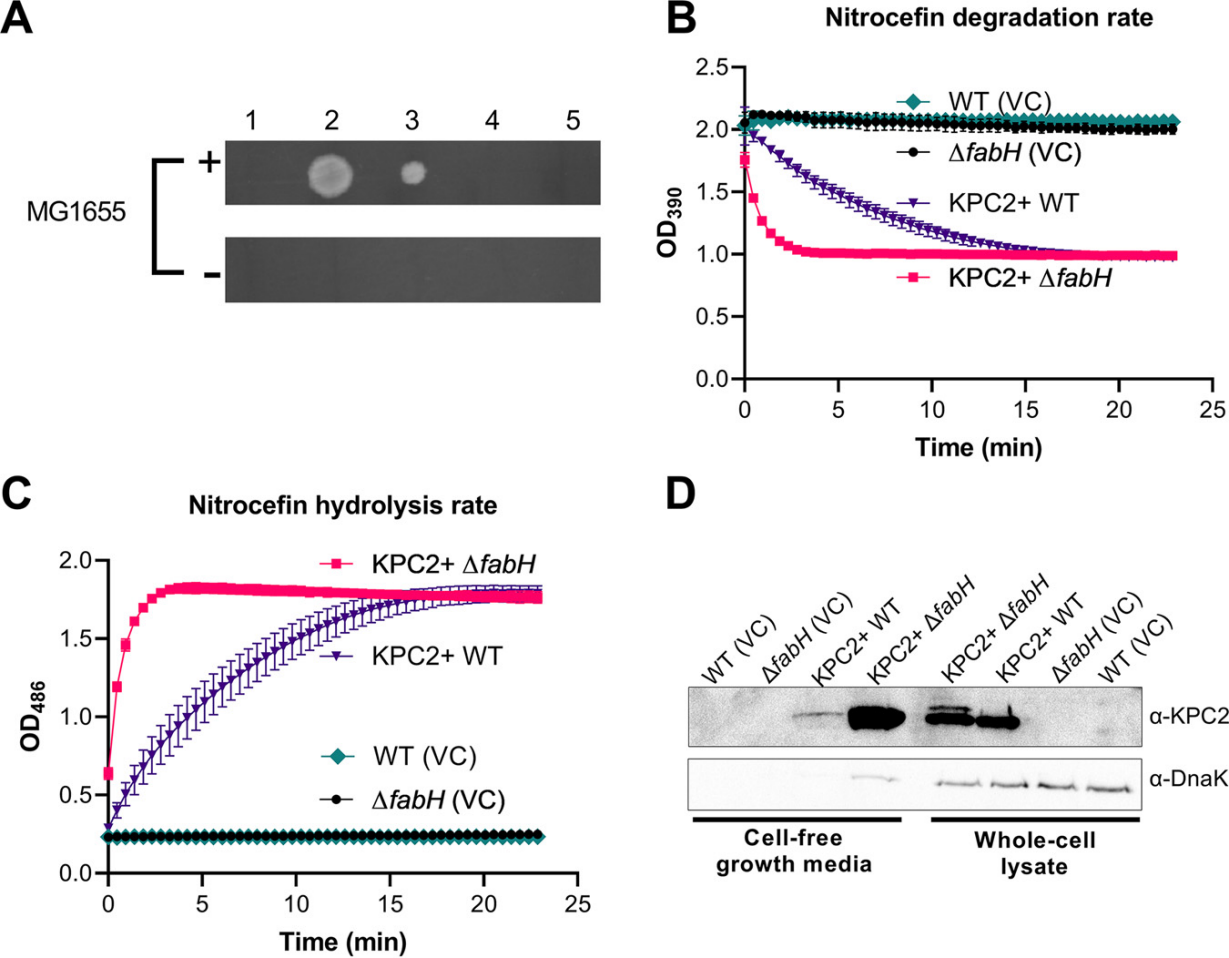
Evidence for leakage from the CFT073 Δ*fabH* severely compromised the outer membrane. (A) KPC2+ Δ*fabH* cell-free growth medium rescues MG1655 growth in meropenem-containing agar. (1) Growth medium harvested from KPC2+ WT; (2) growth medium harvested from KPC2+ Δ*fabH*; (3) heat-treated KPC2+ Δ*fabH* growth medium; (4) proteinase K-treated KPC2+ Δ*fabH* growth medium; (5) LB-Lennox medium control. Cell-free growth media were applied to meropenem plates swabbed with MG1655 (+) or no bacteria (–). (B) Nitrocefin degradation assays using cell-free growth media recovered from KPC2+ CFT073 WT and Δ*fabH*. (C) Nitrocefin hydrolysis assays using KPC2+ CFT073 WT and Δ*fabH* growth medium. (D) Western blot of KPC2 present in cell-free growth media and whole-cell preparations. Data from three biological replicates are shown in panels B and C as means ± SD. Images shown in panels A and D are representative of three biological replicates.

Prompted by the enhanced vulnerability of Δ*fabH* strains to last-line β-lactams and considering the severe OM defects observed in the UPEC strains lacking KAS III activity, we hypothesized that periplasmic β-lactamases may escape from the cell through the compromised OM, thereby reducing the periplasmic concentration of these protective enzymes in the previously resistant strain. To test this tenet, we collected cell-free media from the late log phase cultures of KPC2+ CFT073 WT and Δ*fabH* strains. Medium harvested from the KPC2+ Δ*fabH* culture, but not KPC2+ WT, rescued the growth of carbapenem-susceptible E. coli K-12 MG1655 on LB-Lennox agar containing 4 μg/mL meropenem (Fig. 5A). Proteinase K treatment of this growth medium reversed the growth rescue of MG1655. In contrast, heat treatment only marginally reduced the growth of MG1655 on meropenem LB-Lennox (Fig. 5A). This observation is consistent with the previously reported thermostability of KPC2 (34).

We next diluted cell-free culture media to assay β-lactamase activity with nitrocefin, a chromogenic cephalosporin substrate (Fig. 5B and C) (35). Growth medium harvested from KPC2+ Δ*fabH* exhibited significantly stronger β-lactamase activity than that of the KPC2+ WT (Fig. 5B and C). To confirm that KPC2 was present in significant amounts in the Δ*fabH* cell-free culture medium, proteins from the supernatant were concentrated and analyzed by Western blotting using anti-KPC2 antisera. As expected, we detected KPC2 in whole-cell lysates of KPC2+ CFT073 but not in the vector control (Fig. 5D). KPC2+ CFT073 Δ*fabH* had amounts of KPC2 comparable to those of the WT, although an additional higher-molecular-weight band consistent with premature KPC2 was also detected (Fig. 5D).

In the cell-free culture supernatant fraction, we detected a weak signal for KPC2 in the KPC2+ CFT073 sample (Fig. 5D). In striking contrast, very large amounts of KPC2 were observed in the KPC2+ Δ*fabH* culture medium sample (Fig. 5D). A weak band corresponding to the cytoplasmic chaperone protein DnaK was also detected, indicating minor levels of cell lysis (Fig. 5D). However, based on the very high KPC2:DnaK ratio in the sample compared to that of the whole cell (Fig. 5D), we reasoned that the bulk of KPC2 detected in the cell supernatant likely escaped to the extracellular milieu through the severely compromised OM of Δ*fabH* intact cells.

### Loss of KAS III activity potentiates ceftriaxone treatment of ESBL+ UPEC biofilms and infected human bladder cells.

**FIG 6:**
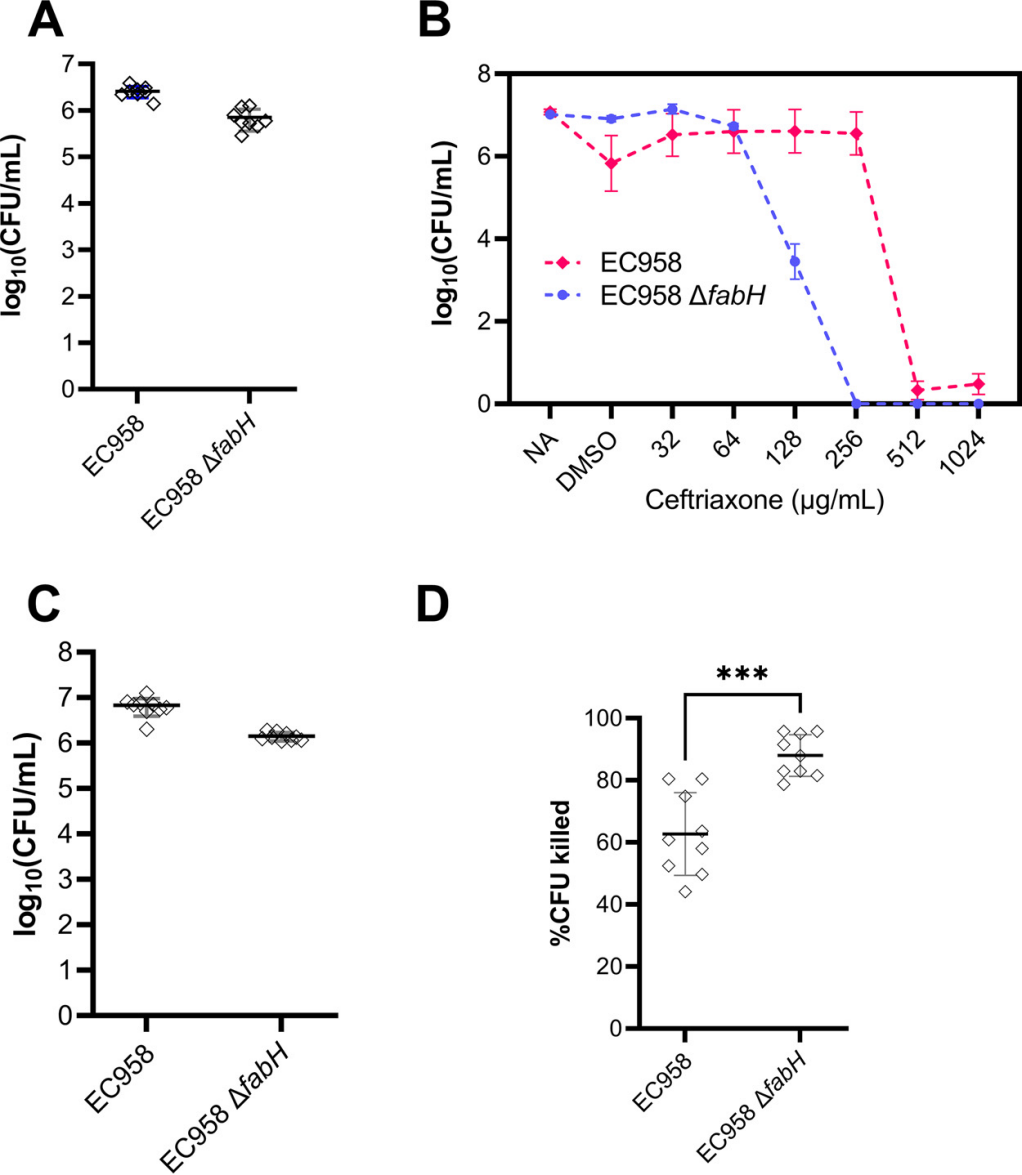
Preclinical evaluation of KAS III as a target aiding the eradication of UPEC biofilms and treatment of human bladder cell infection with antibiotics. (A) EC958 WT and Δ*fabH* establish mature biofilms of comparable biofilm on the Calgary biofilm device; (B) Ceftriaxone MBEC assessment of EC958 WT and Δ*fabH* biofilms, including untreated (NA) and drug carrier (DMSO) controls. (C) Total adherent bacteria on T24 bladder cell monolayers infected for 24 h at an MOI of 10 with EC958 WT and Δ*fabH*. (D) Reduction in viable CFU recovered from UPEC-infected T24 monolayers following a 1-h treatment with ceftriaxone (8 μg/mL). Group means were compared by an unpaired *t* test (***, *P* = 0.003).

UPEC bacteria form biofilms on biotic and abiotic surfaces that are recalcitrant to antibiotic treatment and aid bacterial survival inside bladder cells and on urinary catheters (36, 37). To investigate whether the enhanced antibiotic susceptibility of UPEC Δ*fabH* strains observed in planktonic drug sensitivity assays can be extended to improved activity against established biofilms, we grew EC958 WT and Δ*fabH* mature biofilms with both strains establishing comparable viable biofilm cell densities after 24 h (Fig. 6A). While biofilms formed by either strain had very high ceftriaxone resistance, a 4-fold reduction in the minimal biofilm eradication concentration (MBEC) was observed for Δ*fabH*, with a ceftriaxone MBEC of 512 μg/mL for the WT and 128 μg/mL for Δ*fabH* (Fig. 6B). Interestingly, residual EC958 WT cells remained viable even at the highest ceftriaxone dose tested (1,024 μg/mL), while no viable Δ*fabH* cells were detected in biofilms treated at and above 256 μg/mL ceftriaxone, i.e., achieving complete biofilm eradication (Fig. 6B).

We next tested if ceftriaxone is effective in treating established human cell infection by EC958—a strain that is clinically resistant to this antibiotic. Infection of human T24 bladder cell monolayers with EC958 WT and Δ*fabH* at a multiplicity of infection (MOI) of 10 resulted in high-level adhesion (>10^6^ CFU per monolayer) by both strains, albeit at slightly lower levels for Δ*fabH* (Fig. 6C). Subsequent 1-h treatment of infected monolayers with 8 μg/mL ceftriaxone eliminated ~90% of Δ*fabH* from the monolayer, while only a <65% reduction was observed for the WT (Fig. 6D). Taken together, our preclinical data on improved antibiotic activity against resistant E. coli in planktonic, biofilm, and cell infection models support the tenet that FabH constitutes a promising antimicrobial target for reviving failing last-resort antibiotics.

## DISCUSSION

AMR is one of the top 10 global public health threats of the 21st century, and tackling this invisible pandemic constitutes a current priority (38–40). A recent report highlights that 1.27 million deaths in 2019 were directly attributable to resistant bacterial infections, with E. coli being the leading cause (6). Tactics to extend the life of existing antimicrobial agents or expand their spectrum are becoming a necessity in light of the dwindling antibiotic discovery pipeline, as new antibiotics are sparse. As an evolving permeability barrier, the OM is instrumental in developing antibiotic resistance in Gram-negative bacteria (9, 41). In recent years, there has been a resurgence of interest in exploring the disruption of the OM as an antimicrobial tactic (42–45). FAs are the common building blocks for structural lipids in the cytoplasmic membrane and the OM; thus, their biosynthetic pathways present attractive antibiotic targets (46). Here, we showed that the loss of KAS III incapacitates the OM to drastically improve antibiotic activity against clinical E. coli, even in the presence of acquired antibiotic resistance determinants.

Carbapenem is one of the last-resort antibiotics used to treat infections caused by drug-resistant Gram-negative bacterial infections. Unfortunately, resistance to carbapenems is increasingly prevalent in E. coli and other members of the *Enterobacteriaceae* family (47). We showed that targeting FabH can deactivate the antibiotic protection offered by advanced β-lactamases and carbapenemase to clinical E. coli strains. Another attractive prospect of targeting FabH (or other similar membrane perturbation tactics), is expanding the range of drugs with physicochemical properties amenable to Gram-negative entry, potentially expanding the spectrum of activity of many Gram-positive antibiotics.

FabH was previously thought to be indispensable in E. coli (48). However, this essentiality is bypassed by the product of the *yiiD* gene (renamed MadA) (49). The bypass mechanism involves the decarboxylation of malonyl ACP to produce acetyl ACP (50). Unlike acetyl-CoA, which can only be integrated into the initiation step of FA biosynthesis by FabH, this substrate can be used by FabB/FabF to bypass the loss of KAS III activity in E. coli (50). However, the supply of FA to integrate into membrane biogenesis by the MadA bypass is highly restrictive, and as such, earlier E. coli K-12 studies reported that Δ*fabH* cells are tiny (26, 27). This observation had been instrumental in developing the lipid-centric view that FA availability sets the capacity of the cell envelope to dictate cell size (27). Intriguingly, both CFT073 and EC958 Δ*fabH* cells have comparable size to their corresponding WT cells, though at the expense of cell division rate (Fig. S2). Together, these findings suggest that an additional layer of regulation might be employed in the two UPEC strains, allowing them to reach a destined cell envelope capacity despite severe FA starvation. Nonetheless, we note that antibiotic potentiation of Δ*fabH* is unrelated to cell size, given that K-12 Δ*fabH* cells with reduced size also displayed increased sensitivity to hydrophobic antibiotics (26), and we also observed a 42-fold and 6-fold potentiation to vancomycin and fusidic acid in K-12 strains lacking KAS III (data not shown).

10.1128/msphere.00117-22.5 FIG S2 Flow cytometry analysis of E. coli UPEC wild-type and Δ*fabH* strains. An overnight culture was diluted 40-fold in fresh Cation-Adjusted Mueller–Hinton Broth (CaMHB) and incubated at 37°C until mid-log phase, and then 1 mL of the culture was collected and analyzed in a CytoFLEX S flow cytometer (Beckman Coulter) and with Kaluza Analysis 2.1 (Beckman Coulter, Inc., Brea, CA, USA) by a single user. (A to D) Representative plots showing the cell size (forward scatter) and granularity (side scatter) of (A) EC958 WT, (B) EC958 *ΔfabH*, (C) CFT073 WT, and (D) CFT073 *ΔfabH*. Download FIG S2, TIF file, 0.2 MB. Copyright © 2022 Hong et al. 2022 Hong et al. https://creativecommons.org/licenses/by/4.0/ This content is distributed under the terms of the Creative Commons Attribution 4.0 International license.

Two recent studies had linked FA starvation to increased antibiotic tolerance (51, 52). Sublethal inhibition of FA biosynthesis either by blocking the chain elongation steps catalyzed by FabB/FabF or the enoyl ACP reductase FabI activates the (p)ppGpp synthetase, RelA, which overproduces ppGpp, the effector molecule of stringent bacterial response (51, 52). A high ppGpp level inadvertently drives E. coli and several other species to reach an antibiotic-tolerant state (51–57). The loss of KAS III also triggers the overproduction of ppGpp (26). In fact, the production of the stringent response alarmone is necessary for the survival of Δ*fabH*, as ppGpp is required to drive the transcription of MadA that partially substitutes FabH (49). Yet, in multiple E. coli lineages, Δ*fabH* shows hypersensitivity toward a broad panel of antibiotics.

Yao et al. (26) reported that the membrane lipids of Δ*fabH* contain elevated ratios of unsaturated fatty acids. We expected the cytoplasmic membrane would also be altered in a Δ*fabH* strain. However, In the context of drug diffusion, influx across the cytoplasmic membrane, composed of phospholipids, is orders of magnitude faster than flux across the outer membrane. As such, drug diffusion across the outer membrane is the key rate-limiting step to achieve antibiotic potentiation. Given the extent to which the cell envelope is disrupted in Δ*fabH*, presumably allowing leakage of large periplasmic contents (Fig. 5D), this damage alone is sufficient to overcome antibiotic tolerance induced by the stringent response. Nonetheless, to our surprise, the strain that possessed such a cryptic cell envelope remained highly stable, demonstrated by our failed previous attempts to isolate the then unresolved functional bypass of KAS III activity through permissive conditions (Hong Y and Cronan JE, unpublished). The mystery may lie in the multifaceted network regulating FAS II (58, 59) and other related pathways to compensate for defects in membrane biogenesis (60).

## MATERIALS AND METHODS

### Bacterial strains and growth.

The strains and plasmids used in this study are described in Table 1. The E. coli strains were grown at 37°C in lysogeny broth (LB)-Lennox unless otherwise indicated. For solid media, 15 g/L bacteriological agar was added. Ampicillin was used at 100 μg/mL, chloramphenicol at 17 μg/mL, and gentamicin at 10 μg/mL.

**TABLE 1:** Strains and plasmids used in this work

Strains and plasmids	Detail	Source or reference
Strains		
MG1655	E. coli K-12 strain; F-λ-*ilvG rfb-50 rph-1*	Coli Genetic Stock Centre
MT1776	MG1655 Δ*fabH*::*cat*	This work
CFT073	Pyelonephritogenic E. coli isolate (O6:K2:H1)	69
MT2099	CFT073/pBAD322G	This work
MT1534	CFT073 Δ*fabH*::*FRT*	This work
MT2092	CFT073 Δ*fabH*::*FRT*/pBAD322G-*fabH*	This work
MT534	CFT073 Δ*fabR*::*cat*	This work
MT1427	CFT073 Δ*fadR*::*cat*	This work
MT1439	CFT073 Δ*fadD*::*cat*	This work
MT1496	CFT073 Δ*fabF*::*FRT*	This work
MT1803	CFT073/pKPC2	This work
MT1804	CFT073 Δ*fabH*::*FRT*/pKPC2	This work
EC958	E. coli ST131 urinary tract infection isolate (O25b:H4), MDR	23
MT2098	EC958/pBAD322G	This work
MT1667	EC958 Δ*fabH*::*cat*	This work
MT2100	EC958 Δ*fabH*::*cat*/pBAD322G-*fabH*	This work
MT1901	EC958 Δ*fabR*::*cat*	This work
MT1902	EC958 Δ*fabF*::*cat*	This work
MT1903	EC958 Δ*fadD*::*cat*	This work
MT1917	EC958 Δ*fadR*::*cat*	This work
MT1608	EC958 Δ*tolC*::*cat*	This work
Plasmids		
pKPC2	Tn-4401a-*kpc2* cloned into pSU2718, chloramphenicol resistance	This work
pBAD322G	*araBAD* promoter-based expression vector, complete pBR322 origin, gentamicin resistance	28
pSU2718	p15A-derived plasmids carry *lacZα* reporter gene, chloramphenicol resistance	33
pBAD322G-*fabH*	K-12 MG1655 *fabH* cloned into the BamHI/PstI sites of pBAD322G	This work
pKD3	FRT-flanked cat gene, oriRγ replicon, ampicillin, and chloramphenicol resistance	62
pKD46	λ recombineering genes (α, β, γ) controlled by arabinose-inducible promoter, P*araB*, temp-sensitive oriR101 replicon, ampicillin resistance	62
pCP20	*flp* recombinase gene, temp-sensitive replicon, ampicillin, and chloramphenicol resistance	62
pKOBERG-Gen	Gentamicin-resistant plasmid carrying the λ recombineering genes (α, β, γ)	70

### Cloning and genetic manipulations.

The *fabH* gene was amplified from the genomic DNA of the K-12 strain MG1655 and cloned into the BamHI and PstI sites in the pBAD322G vector (28). pKPC2 was constructed by amplifying Tn*4401a*-*kpc2* from the genomic DNA of a clinical K. pneumoniae strain, JIE2709 (61), and cloned into the PstI and KpnI sites in pSU2718 (33). Lambda recombination competency was achieved by adding temperature-sensitive plasmids, either pKD46 (62) or pKOBERG-Gen (63), to the manipulating strain. Transformation and lambda recombination were performed as previously described (64). Temperature-sensitive pCP20-Gent carrying FLP recombinase was used to remove the *cat* marker wherever applicable. For the construction of EC958 Δ*tolC*::*cat*, lambda-red recovery culture postelectrotransformation was platted onto a low-dose chloramphenicol (6.5 μg/mL chloramphenicol) LB-Lennox plate and isolated and screened from a thin bacterial lawn. Oligonucleotide sequences used for gene replacements and cloning are included in Table S1.

10.1128/msphere.00117-22.1 TABLE S1 Oligonucleotides used in this study. Download Table S1, DOCX file, 0.01 MB. Copyright © 2022 Hong et al. 2022 Hong et al. https://creativecommons.org/licenses/by/4.0/ This content is distributed under the terms of the Creative Commons Attribution 4.0 International license.

### Outer membrane defect assay.

Vancomycin can only transverses the Gram-negative OM when the permeability barrier is compromised (65). The OM defect of the null mutants constructed was assayed using susceptibility of a dilution series from overnight-grown cultures adjusted to an OD_600_ of 1.0 to subinhibitory concentrations of vancomycin similar to the method previously described (66, 67). Equivalent loading of the OD_600_-adjusted samples (5 μL) was spot-plated onto LB agar.

### Ethidium bromide efflux assay.

Stationary cultures were diluted with fresh LB-Lennox containing 1 mg/L ethidium bromide and 20 μg/mL carbonyl cyanide *m*-chlorophenylhydrazone. Cultures were incubated at 37°C until an OD_600_ of 0.7 to 0.8 was reached with aeration. The cells pellets were harvested via centrifugation and washed with 4× volumes of 1× phosphate-buffered saline (PBS). The samples were then resuspended in 1× PBS and incubated for 30 min in a dark room at 5°C. The samples were adjusted to an OD_600_ of 0.2 and dispensed in 150-μL volumes in a Greiner 96-well flat-bottom plate; d-glucose (20% wt/vol stock) was then added at a 0.1% concentration (12 μL) to energize ethidium bromide efflux. Efflux activity was tracked over 60 min in a CLARIOStar instrument (BMG, Australia) at 37°C. The following instrumental settings were used: excitation at 525 ± 15, emission at 615 ± 20, auto 568.8 dichroic filter. The experiment was performed in biological triplicates.

### Determination of antibiotic MICs.

The specific bacterial strains were prepared and tested according to the MIC test strips (Liofilchem) manufacturer’s instructions. Briefly, overnight cultures of the specific strains were diluted in 0.85% (wt/vol) saline to give a final inoculum concentration of 1.5 × 10^8^ CFU mL^−1^. The inoculums were then used to inoculate Mueller-Hinton agar (Thermo Fisher, Australia) plates using a sterile swab. The MIC test strip was applied to the agar surface after the inoculum had dried. Plates were then incubated at 37°C for 20 h. The MIC values were determined by observing where the relevant inhibition ellipse intersected with the MIC test strip.

### Determination of carbapenemase activity in cell-free growth media using MG1655 rescue.

MT1803 and MT1804 overnight early stationary cultures were diluted 1:50 into fresh LB-Lennox containing chloramphenicol (17 μg/mL) and grown to an OD_600_ of 0.8 at 37°C. Cell-free growth media were prepared by the filtration of culture supernatant of OD_600_ 0.8 cultures grown at 37°C with aeration through a 0.22-μm filter (3M). The samples (5 μL) were spotted onto LB-Lennox agar containing meropenem (4 μg/mL) and had been swabbed with overnight cultures of MG1655 on the plate surface. Cell-free growth media treated with proteinase K at 37°C for 30 min and heat-treated (60°C for 30 min) cell-free growth media were also added to MG1655-swabbed LB-Lennox containing meropenem (4 μg/mL) to serve as controls. LB-Lennox containing meropenem (4 μg/mL) without MG1655 swab was also included as a control for the cell-free growth media. Plates were incubated at 37°C for 20 h, and images were taken with a Bio-Rad GelDocXR+ system using Image Lab software (v5.1; Bio-Rad). Figure 5D is representative of three biological replicates.

### Assaying carbapenemase activity from cell-free growth media.

MT1803 and MT1804 overnight early stationary cultures were diluted 1:50 into fresh LB-Lennox containing chloramphenicol (17 μg/mL) and grown to an OD_600_ of 0.8 at 37°C. The cell-free growth medium was prepared by filtering culture supernatant through a 0.22-μm filter (3MM). The cell-free growth media were then diluted 1:1,000 (180 μL) and adjusted to 150 μM nitrocefin to give a testing volume of 200 μL. Nitrocefin degradation assays using cell-free growth media recovered from KPC2+ CFT073 WT and Δ*fabH* were then performed using KPC2+ CFT073 WT and Δ*fabH* growth medium, with two variables, degradation of nitrocefin and generation of products tracked at 390 nm and 486 nm, respectively, on a CLARIOStar instrument (BMG, Australia) over time. Nitrocefin working stock was prepared at 1.5 mM in 50 mM phosphate buffer.

### Precipitation of extracellular proteins and Western blot analysis.

First, 5 mL of cell-free growth medium was prepared by the filtration of culture supernatant of OD_600_ 0.8 cultures grown at 37°C with aeration through a 0.22 μm filter (3MM). The sample was then precipitated with 1 mL of ice-cold 100% (wt/vol) trichloroacetic acid and incubated on ice for 10 min. The precipitated protein was harvested via centrifugation (20,000 × *g*, 4°C, 10 min), washed once with 500 μL of ice-cold acetone, and dissolved in 100 μL of SDS-PAGE sample buffer, with the pH adjusted with 1 M Tris-HCl, pH 9. Precipitated extracellular protein samples (10 μL) and whole-cell lysates (10 μL, derived from a 10× concentrate of OD_600_ 1.0 culture harvested simultaneously as cell-free growth media) were separated on 12% SDS-PAGE. The samples were transferred to nitrocellulose membranes. Immunoblot analyses were performed with rabbit α-KPC2 (1:8,000) and mouse α-DnaK (1:10,000; Enzo Life Sciences, kindly gifted by Renato Morona), followed by goat α-rabbit Amersham ECL HRP-conjugated antibodies (1:20,000; Cytiva) for chemiluminescent detection using Pierce ECL Western blot substrate. Rabbit polyclonal α-KPC2 sera were raised by inoculation with three previously described KPC2 peptide epitopes (epitope A: CFAKLEQDFGGSIGVYA; epitope B: CLNSAIPGDARDTSSPRAVT; epitope C: CVIAAAARLALEGLGVN [68]) at the Walter and Eliza Hall Institute of Medical Research (peptides were synthesized by Mimotopes Pty. Ltd.). The developed blots were imaged in the Bio-Rad GelDocXR+ system using Image Lab software (v5.1; Bio-Rad).

### Biofilm growth.

Minimum biofilm eradication concentration (MBEC) and biofilm growth assays were performed in a Calgary biofilm device (CBD) (MBEC assay; Innovotech, Inc., Canada). Overnight bacterial cultures in LB were diluted to 10^6^ CFU/mL and used to inoculate the plate with 130 μL of culture per well. The CBD was incubated for 24 h with shaking (150 rpm) at 37°C in 95% relative humidity. Following 24 h of growth, biofilms were washed once in PBS to remove nonadherent cells and then sonicated for 20 min at 20°C. At least three biological and two technical repeats per strain per experiment were serially diluted and spotted onto LB agar plates to determine viable CFU recovered from each peg biofilm. Plates were incubated overnight at 37°C, and colonies were counted the following day to obtain log_10_ (CFU/mL) values for each strain.

### MBEC assays.

Following 24 h of biofilm growth in the CBD, biofilms were washed once in PBS to remove nonadherent cells, and the biofilm peg lid was transferred to a treatment plate containing various concentrations of ceftriaxone disodium salt heptahydrate or dimethyl sulfoxide (DMSO) in Muller Hinton broth. Peg biofilms were incubated in the treatment plate for 24 h and then sonicated and plated as per the biofilm growth assay. MBEC values were defined as concentrations with over 3 log_10_ reduction in CFU/mL.

### Epithelial cell infection assay and ceftriaxone treatment.

Intestinal epithelial T24 cells (ATCC HTB4; Dulbecco’s modified Eagle’s medium [DMEM]) were maintained in McCoy medium (Invitrogen) supplemented with 5% heat-inactivated fetal calf serum (Invitrogen). Bacterial strains were cultured under type 1 fimbria enrichment conditions, as previously described (23). Infection assays were performed as previously described (63). Briefly, confluent cell monolayers were infected with strains at a multiplicity of infection (MOI) of 10 and incubated at 37°C, 5% CO_2_, for 1 h. PBS washes (3×) were used to remove nonadherent bacteria. Ceftriaxone salt heptahydrate (in DMSO) was added to McCoy medium and applied to the infected monolayer without disturbance. Antibiotic treatment was performed by incubation at 37°C, 5% CO_2_, for 2 h. Then, 1× PBS washes (3×) were used to remove nonadherent bacteria and antibiotic residue. Monolayers were lysed with 0.1% (vol/vol) Triton X-100, and lysates were serially diluted and plated onto LB agar to enumerate total adherent bacteria.

10.1128/msphere.00117-22.6 FIG S3 Fluorescence microscopy showing the cell size of UPEC wild-type and Δ*fabH* strains. An overnight culture was diluted 40-fold in fresh CaMHB and incubated at 37°C until mid-log phase, and then 1 mL of the culture was collected and washed with Hank’s balanced salt solution. The sample was resuspended in FM4-64FX (5 μg/mL) and chilled on ice for 5 min. Cells were then washed once with Hank’s balanced salt solution, and samples were observed using and inverted LSM 880 fast Airyscan microscope. Images shown are representative fields of view obtained from two biological repeats. Download FIG S3, TIF file, 1.3 MB. Copyright © 2022 Hong et al. 2022 Hong et al. https://creativecommons.org/licenses/by/4.0/ This content is distributed under the terms of the Creative Commons Attribution 4.0 International license.
