# Supplementary figures and images for: Loss of β-Ketoacyl Acyl Carrier Protein Synthase III Activity Restores Multidrug-Resistant Escherichia coli Sensitivity to Previously Ineffective Antibiotics
Source: mSphere. 2022 May 16;7(3):e00117-22. doi: 10.1128/msphere.00117-22 (PMC9241538; doi:10.1128/msphere.00117-22)

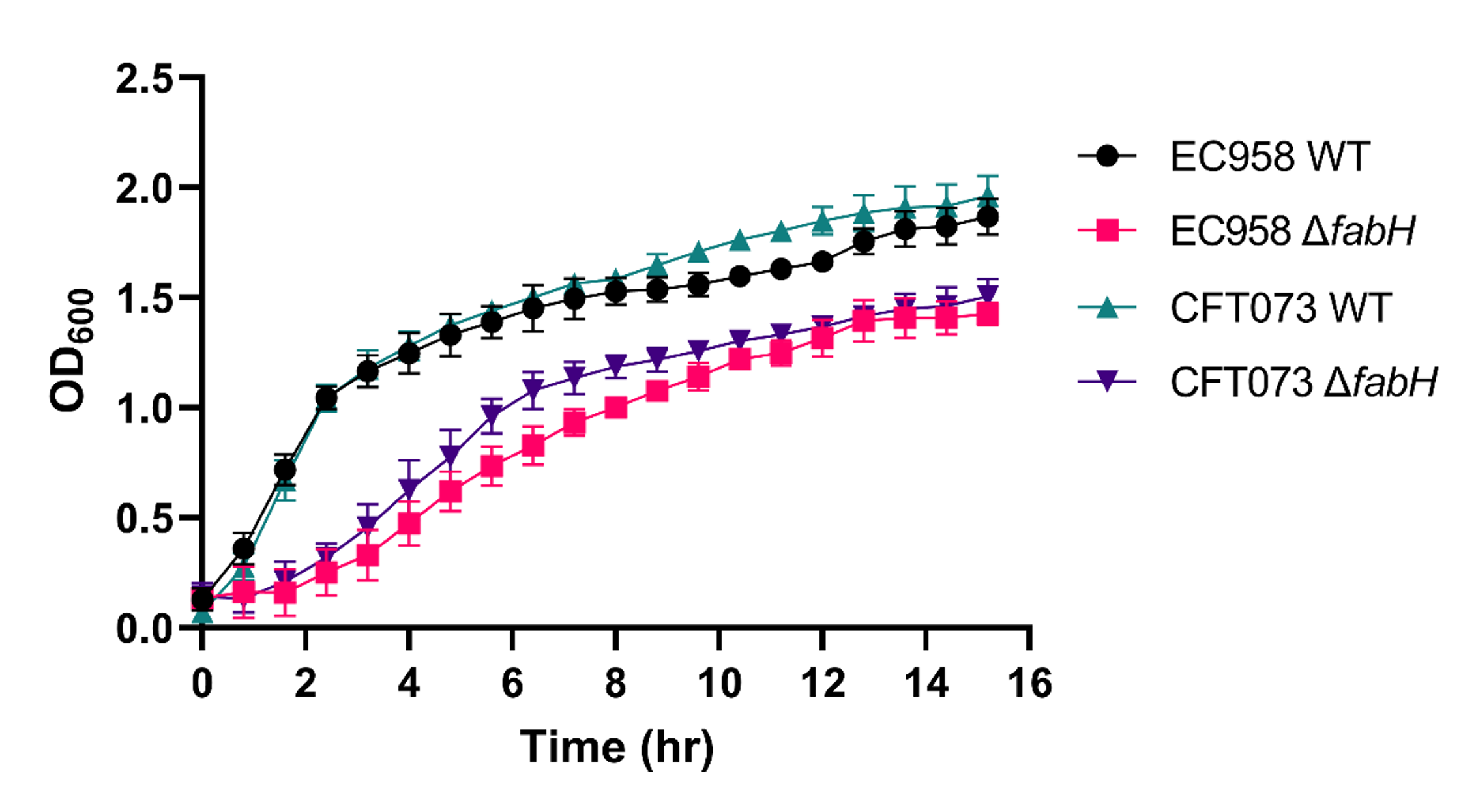

Supplement: FIG S1 [file msphere.00117-22-s0004.tif]

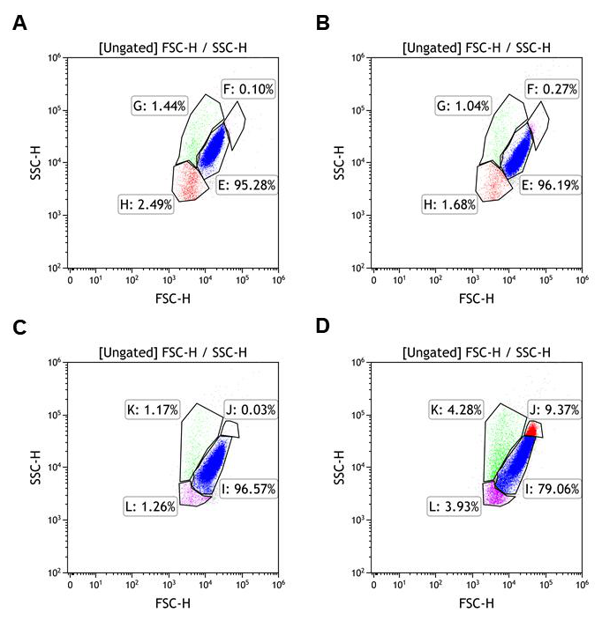

Supplement: FIG S2 [file msphere.00117-22-s0005.tif]

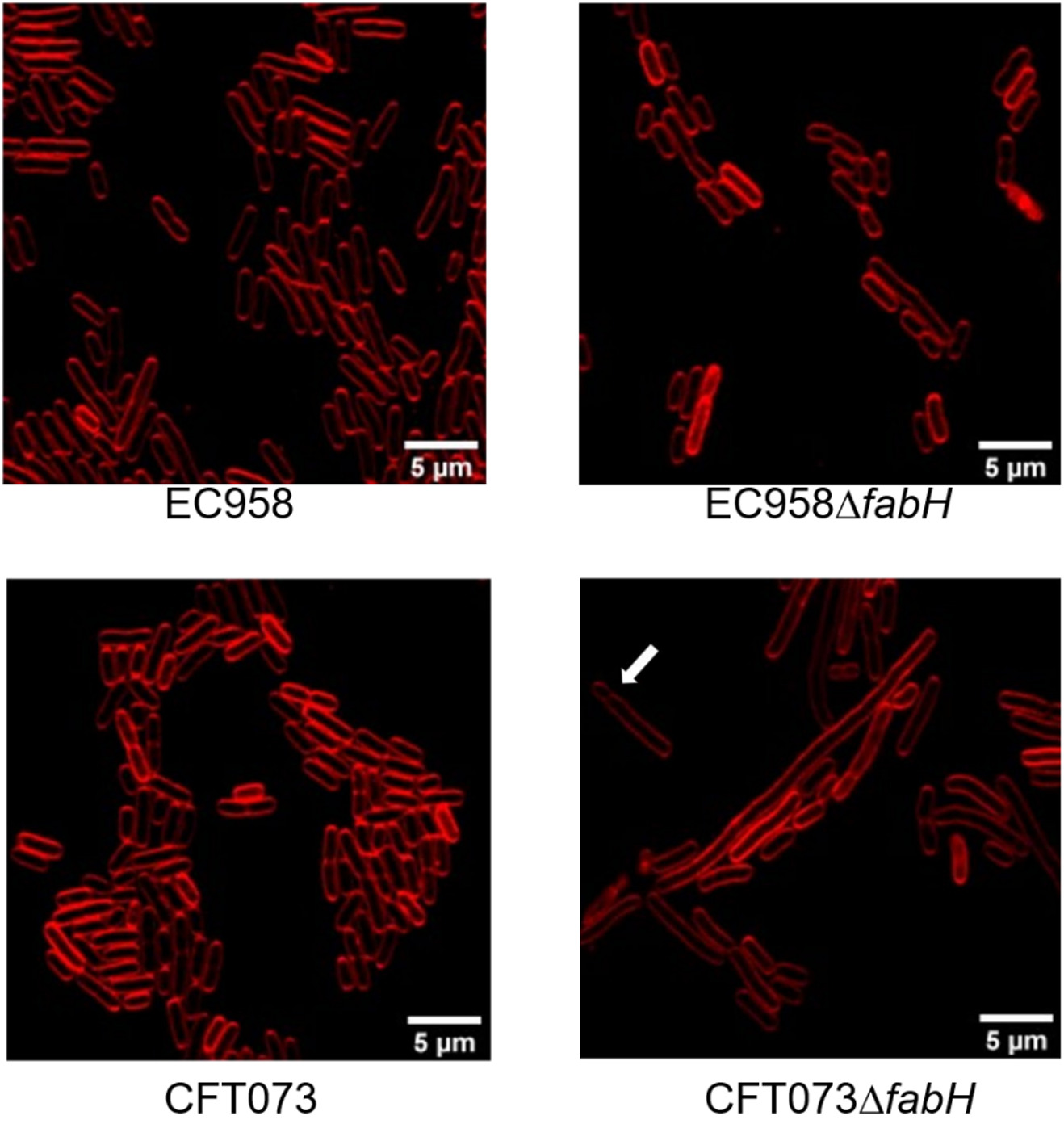

Supplement: FIG S3 [file msphere.00117-22-s0006.tif]
