# Supplementary material for: Loss of β-Ketoacyl Acyl Carrier Protein Synthase III Activity Restores Multidrug-Resistant Escherichia coli Sensitivity to Previously Ineffective Antibiotics
Source: mSphere. 2022 May 16;7(3):e00117-22. doi: 10.1128/msphere.00117-22 (PMC9241538; doi:10.1128/msphere.00117-22)
Supplement: TABLE S2 [file msphere.00117-22-s0002.docx]

| Antibiotics | MIC-value (*µ*g mL^-1^) | | | |
| --- | --- | --- | --- | --- |
|  | CFT073 | CFT073 ∆*fabH* | EC958 | EC958 ∆*fabH* |
| Kanamycin | 2 | 0.19 | >32 | 12 |
| Gentamicin | 0.75 | 0.064 | 1.5 | 0.38 |
| Amikacin | 1.5 | 0.19 | 12 | 4 |
| Tobramycin | 2 | 0.25 | 24 | 8 |
| Ciprofloxacin | 0.006 | 0.002 | >32 | >32 |
| Co-trimoxazole | 0.064 | 0.064 | >32 | >32 |
| Azetreonam | 0.064 | 0.064 | 3 | 0.064 |
| Ampicilin | 2 | 0.38 | >256 | >256 |
| Ceftriaxone | 0.016 | 0.004 | 16 | 0.5 |
| Meropenem | 0.012 | 0.003 | 0.012 | 0.003 |
| Colistin | 2 | 0.75 | 2 | 1 |
| Vancomycin | >256 | 4 | >256 | 8 |
| Fusidic acid | >256 | 32 | >256 | 32 |
| Chloramphenicol | 8 | 1 | NA | NA |
| NA: Not assessed. | | | | |
