# Supplementary material for: Loss of β-Ketoacyl Acyl Carrier Protein Synthase III Activity Restores Multidrug-Resistant Escherichia coli Sensitivity to Previously Ineffective Antibiotics
Source: mSphere. 2022 May 16;7(3):e00117-22. doi: 10.1128/msphere.00117-22 (PMC9241538; doi:10.1128/msphere.00117-22)
Supplement: TABLE S3 [file msphere.00117-22-s0003.docx]

| Antibiotics | MIC-value (*µ*g mL^-1^) | |
| --- | --- | --- |
|  | KPC2+ CFT073 | KPC2+ CFT073 ∆*fabH* |
| Meropenem | 32 | 1.5 |
| Imipenem | 32 | 1 |
| Doripenem | 6 | 0.19 |
| Aztreonam | >1024 | 24 |
