# Supplementary material for: Loss of β-Ketoacyl Acyl Carrier Protein Synthase III Activity Restores Multidrug-Resistant Escherichia coli Sensitivity to Previously Ineffective Antibiotics
Source: mSphere. 2022 May 16;7(3):e00117-22. doi: 10.1128/msphere.00117-22 (PMC9241538; doi:10.1128/msphere.00117-22)
Supplement: TABLE S1 [file msphere.00117-22-s0001.docx]

| **Primer Name** | **Sequence (5'-3')** |
| --- | --- |
| EcfabF_KO_Fw | AGGCCCGCAAGCGGACCTTTTATAAGGGTGGGAAATGACAACGTGTAGGCTGGAGCTGCTTC |
| EcfabF_KO_Rv | TTGTCCCACTAGAATCATTTTTTCCCTCCCTGGAGGACAAACCCTATTCCGAAGTTCCTA |
| EcfabH_KO_Fw | AGGGAACACAAATGCAAATTGCGTCATGTTTTAATCCTTATCGTGTAGGCTGGAGCTGCTTC |
| EcfabH_KO_Rv | TTTGCAAGTGACGGTATATAACCGAAAAGTGACTGAGCGTACCCTATTCCGAAGTTCCTA |
| EcfabR_KO_Fw | GCTAGCAGCAGCGTACCTCTATCTTGATTTGCTTGTTTCATGTGTAGGCTGGAGCTGCTTC |
| EcfabR_KO_Rv | TTATTGCGTTACCGTTCATTCACAACACTGGAGCAATCCAGTCCTATTCCGAAGTTCCTA |
| EcfadR_KO_Fw | ATACGTTTGTCATCCGTCTGGAAGGATTATCGTCCCTGAATGGTGTAGGCTGGAGCTGCTTC |
| EcfadR_KO_Rv | TGAGTCCAACTTTGTTTTGCTGTGTTATGGAAATCTCACTATGGGAATTAGCCATGGTCC |
| EcfadD_KO_Fw | ACTGACTTAACGCTCAGGCTTTATTGTCCACTTTGCCGCGCGTGTAGGCTGGAGCTGCTT |
| EcfadD_KO_Rv | TCCTTGGGTAATTATCAAGCTGGTAAGATGAGTTAATATTATGGGAATTAGCCATGGTCC |
| EcTolC_KO_Fw | CCTTTTGCGGTAGCGGCTTCTGCTAGAATCCGCAATAATTTTACAGTGTAGGCTGGAGCTGCTTC |
| EcTolC_KO_Rv | CTCGCTGGCACCAACAAAGTTGTACTGGGCCTGTTTCACCATGGGAATTAGCCATGGTCC |
| MG1655_fabH_Fw_BamHI | ATCGAGGATCCATGTATACGAAGATTATTG |
| MG1655_fabH_Rv_PstI | AGCTAACTGCAGCTAGAAACGAACCAGCGCG |
| Tn4401-kpc_Fw_PstI | AACGACTGCAGGCCAATAGATGATTTTCAGAGCC |
| Tn4401-kpc_Rv_KpnI | AGCAAGGTACCCAGGGGTAAAGTGGGTCAG |
